# Grand Challenges in Human Factors and Digital Health

**DOI:** 10.3389/fdgth.2021.635112

**Published:** 2021-05-24

**Authors:** Stephen M. Schueller

**Affiliations:** ^1^Department of Psychological Science, University of California, Irvine, Irvine, CA, United States; ^2^Department of Informatics, University of California, Irvine, Irvine, CA, United States

**Keywords:** digital health, grand challenge, mHealth, design, methodology

## Introduction

We live in a world where the potential of technology continues to expand. Every year computers become more powerful, smaller, and cheaper, and the amount of data created and stored increases. Technology is quickly entering and transforming various areas of our life, and health and healthcare are no exceptions. However, many of the promises offered by digital health—personalized care, scalable interventions, cost-effective approaches, reduced disparities (1)—are unmet. Although it has been noted that digital health can contribute to the so-called triple aim of health care reform (2)—improved patient experience, population health, and cost—healthcare often remains disjointed, significant health problems and health disparities exist, and healthcare costs continue to increase. Thus, digital health remains high on promise and potential, but low on impact and benefit, and yet research on digital health continues to expand. Much of this research, however, is problematic. A review of clinical trials of digital health found rapid growth but most studies were small with few reporting findings even among those completed or terminated (3). Thus, we have an urgent need to revamp digital health research to ensure the promise and potential of this field can be realized.

Increased consideration of human factors offers the potential to improve and advance digital health research and maximize its impact in the various fields that contribute to this work, and on people's lives. Human factors reference human emotions, behaviors, and cognitions related to the design, adoption, usage, and implementation of health technologies. As such, human factors and digital health lie at the intersection of several areas of scholarship including clinical science, human-computer interaction, implementation science, public health, and healthcare communication. Although many researchers and providers are approaching the same problems from different fields, we have an increased need to recognize ways to better work together to reach mutual goals. For example, the appreciation that the study of digital health requires addressing issues beyond the technology itself and addressing socio-technical considerations (4) aligns strongly with the socio-ecological model emphasized by the consolidated framework for implementation research (5). As such, I suggest that one grand challenge facing research related to human factors and digital health is that we have to re-think this area of study by adopting three critical reframes.

## Three Critical Reframes in Digital Health

### Digital Health and Digital Health Interventions Are Not Products, They Are Instead Technology-Enabled Services

Health and healthcare are deeply human topics and therefore digital health should not focus on the products but should instead focus on the new connections and new services that such technologies have enabled (6). Consideration of human factors shifts the focus to the human components that lie between and within the technologies, understanding what people need and how people want to use technologies by adopting participatory design approaches. Investigating the impact of people's lives and the provider's workflow by conducting real-world deployment studies addressing various stakeholders, and moving beyond purely clinical endpoints to consider user perspectives that impact adoption and usage, abandonment, and sustainment. It is not sufficient to understand what the technology does, but it is important to appreciate the services and interactions it creates.

### Our Research Efforts Should Not Only Generate Knowledge, They Should Solve Problems

The fact that most of our research findings do not impact practice, and even those that do take, on average, 17 years to move from research to practice (7), has been referred to as the research-to-practice gap. Therefore, although research is successful at generating knowledge, it often fails to solve problems and fails to become relevant to people's lives. One reason for these failures is our reliance on traditional phases of discovery, pilot, efficacy, effectiveness, and occasionally, implementation, in the digital health space, as these phases are familiar to clinical science. Instead, we have proposed elsewhere, that phases of create, trial, and sustain, might be more appropriate when it comes to digital health (8). This proposal aligns with other suggestions to use development and evaluation frameworks that blend methods for clinical science and human-centered design (9, 10).

Reframing the research endeavor as solution-focused and adopting the phases of create, trial, and sustain provides opportunities to enhance the applications of of human factors in digital health research. The create phase should be influenced by best practices in human-centered design, ensuring that all stakeholders who will be impacted by a technology have opportunities to contribute to its development. The trial phase needs to move beyond clinical endpoints to consider outcomes relevant to human factors including usability usefulness, or clinical utility. Furthermore, trialing should include understanding not only whether a digital health intervention works but why it works drawing on perspectives from key stakeholders including, but not limited to, patients, providers, and payers. Lastly, we must appreciate that the publication should not be the end phase of research, instead we should be driving toward the sustainable implementation of digital health. This might encourage more works to look at issues stemming from real-world deployments of digital health including considerations of negative or unattended consequences. We also need to strive toward ensuring we do not leave settings or people without some takeaway from the research we conduct with them, be that sustainable technologies themselves or lessons learned relevant to those settings and people rather than just the research endeavor. If we produce scientific publications but fail to solve problems we have failed to meet our goals.

### Digital Health Should Not Be Multidisciplinary or Interdisciplinary, It Should Be a Transdisciplinary

Multidisciplinary work occurs when contributors come from diverse fields but remain siloed by their disciplinary boundaries. Interdisciplinary work occurs when contributors from diverse fields synthesize across fields to produce coordinated work. Transdisciplinary work, however, begins to create new areas of study by transcending traditional boundaries ((11)). Traditional research methodologies and the conceptual models that resulted from those methodologies are not sufficient in the field of digital health. We can collect more data, faster, and in more novel ways than was possible in past research. Ecological momentary assessments, digital phenotyping, and ubiquitous computing give way to idiographic analyses and interventions including personalized medicine and just-in-time adaptive interventions (JITAIs). Furthermore, we need to teach practitioners and researchers new areas of knowledge. Providers who are trained in artificial intelligence and machine learning, clinical researchers who appreciate human-centered design, and technologists who appreciate clinical evidence paradigms and ethics. These silos will not be easy to break down, but one step toward doing so will be increasing collaboration and dialogue between these diverse groups tackling similar problems.

Elsewhere I have noted that many of our digital health interventions are filled with skeuomorphs (1). A skeuomorph is an ornamental remnant of something that was functionally necessary in a previous version. Conceptually a skeuomorph may result when we fail to appreciate that the new affordances of technologies allow us to do things differently rather than recreating the past in digital forms or holding to our traditional practices when technology allows new possibility of methodologies, services, and evaluation. These three reframes I offer are a call to shed skeuomorphic thinking in the digital health space to imagine the possible rather than create the conventional. However, even by tackling these reframes many open questions remain in digital health which form additional challenges that must be overcome to help it reach its promise.

## Where and When Will Care Occur?

Digital health allows opportunities for new models of care delivery that can move assessment, intervention, and prevention outside of traditional clinical settings and into the context of people's daily lives. However, digital health can occur in a variety of places. Technologies can enter into traditional clinical settings to make the work down their more efficient and impactful. It can help bridge clinical settings and people's lives creating “brick-and-click” services that meld traditional services with technology outreaches. They can also circumvent traditional care pathways altogether, creating patient-centric care.

Based on all these possibilities, research must address where and when digital health will be most useful. Such considerations require a consideration of human factors to determine not only the impact on clinical endpoints but also potential benefits across the workflows and lives of patients and providers. The areas of patient-generated health data and personal informatics provide examples of such considerations. For example, an evaluation of various projects related to integrating patient-generated health data into the clinical setting identified three main benefits: (1) deeper insight into conditions, (2) more accurate information, and (3) information gained from real-world settings, between clinics visits; as well as three areas of consideration: (1) developing workflows and protocols to integrate PGHD in practice, (2) promoting data storage and access at point of care, and (3) simplifying the use of PGHD (12). People have to be able to make use of such data in their lives and this requires not only understanding how best to collect and present data but understanding the processes people undertake with such data. Such processes include deciding, selecting, collecting, integrating, and reflection—as well as lapsing and resuming (13). These are deeply human concerns and considering these opportunities for digital health without addressing concerns of human factors will minimize the impact of such work.

## How Do We Approach Digital Health In a Way That Eliminates Rather That Increases Health Disparities and Inequities?

Our current moment in the COVID-19 pandemic has demonstrated the grand challenge of leveraging the use of technology while ensuring that such use does not further exacerbate disparities and inequities. Despite the desire to use digital health in ways to reach those underserved by traditional health services, these populations have been largely left out of the design, deployment, and evaluation of digital health. Digital health offers the potential for deeply personalized interventions but the fact remains that ZIP code alone provides useful information that predicts various health outcomes including mortality (14). Despite reports that technologies are ubiquitous, significant disparities still exist to sufficient Internet and device access that cuts across rural, socio-economic, and racial and ethnicity lines. We have to appreciate that although technologies can often scale, they will only be relevant and useful for diverse groups if we design them to be so and we can only ensure they will be effective if we evaluate whether that is the case.

## How Do We Support Trust in Digital Health Tools We Build?

Digital health tools will be useless if people will not use them, and people will not use them if such technologies do not foster trust. I alluded to various opportunities for digital health, including ecological momentary assessments, digital phenotyping, and ubiquitous computing, such opportunities rely deeply on the willingness of people to contribute to them, by answering questions in the flow of their day, by allowing devices to passively and continuously monitor them, and to carry and wear various devices. Many of the large technology companies have made a business out of grabbing our attention and pushing advertising. Digital health interventions sometimes follow this model, gathering information and passing it to various third-parties (15). Data exploitation is one factor that damages trust, but building trust requires considering personal, institutional, and technological elements (16). Trust is a particularly important area to consider where people, systems, and technologies intersect in digital health, in an effort to ensure digital health is not just useful but that people will actually engage and use it.

## Conclusion

I am excited to see where the field of digital health goes because I truly believe in its potential. I am also excited to see how the work in *Human Factors and Digital Health* helps drive the field forward as I believe there is a gap in venues that truly integrate the diverse work in this area. That is not to say that good work related to human factors in digital health is not being done, but rather that researchers coming from the clinical- and technology-side need opportunities to come together, revisit and reframe their assumptions, address these grand challenges, and drive the field toward better research and better health.

## Author Contributions

The author confirms being the sole contributor of this work and has approved it for publication.

## Conflict of Interest

SS has received payments for consulting to Otsuka Pharmaceuticals unrelated to the content of this manuscript. SS also serves on the Scientific Advisory Board for Headspace for which he receives compensation.
